# Streamlined, Inexpensive 3D Printing of the Brain and Skull

**DOI:** 10.1371/journal.pone.0136198

**Published:** 2015-08-21

**Authors:** Jason S. Naftulin, Eyal Y. Kimchi, Sydney S. Cash

**Affiliations:** 1 Department of Neurology, Massachusetts General Hospital, Boston, Massachusetts, United States of America; 2 Harvard Medical School, Boston, Massachusetts, United States of America; UGent / VIB, BELGIUM

## Abstract

Neuroimaging technologies such as Magnetic Resonance Imaging (MRI) and Computed Tomography (CT) collect three-dimensional data (3D) that is typically viewed on two-dimensional (2D) screens. Actual 3D models, however, allow interaction with real objects such as implantable electrode grids, potentially improving patient specific neurosurgical planning and personalized clinical education. Desktop 3D printers can now produce relatively inexpensive, good quality prints. We describe our process for reliably generating life-sized 3D brain prints from MRIs and 3D skull prints from CTs. We have integrated a standardized, primarily open-source process for 3D printing brains and skulls. We describe how to convert clinical neuroimaging Digital Imaging and Communications in Medicine (DICOM) images to stereolithography (STL) files, a common 3D object file format that can be sent to 3D printing services. We additionally share how to convert these STL files to machine instruction gcode files, for reliable in-house printing on desktop, open-source 3D printers. We have successfully printed over 19 patient brain hemispheres from 7 patients on two different open-source desktop 3D printers. Each brain hemisphere costs approximately $3–4 in consumable plastic filament as described, and the total process takes 14–17 hours, almost all of which is unsupervised (preprocessing = 4–6 hr; printing = 9–11 hr, post-processing = <30 min). Printing a matching portion of a skull costs $1–5 in consumable plastic filament and takes less than 14 hr, in total. We have developed a streamlined, cost-effective process for 3D printing brain and skull models. We surveyed healthcare providers and patients who confirmed that rapid-prototype patient specific 3D models may help interdisciplinary surgical planning and patient education. The methods we describe can be applied for other clinical, research, and educational purposes.

## Introduction

Neuroimaging technologies such as Magnetic Resonance Imaging (MRI) and Computed Tomography (CT) have become indispensable tools for the diagnosis and treatment of central nervous system disease. While both MRIs and CTs collect three-dimensional data (3D), clinicians typically view the data on two-dimensional (2D) computer monitors. 2D representations limit our appreciation of complex surfaces, such as brain convolutions or skull curves. Although software exists to make virtual 3D models of internal organs, 2D display of these models on computer monitors prevents us from being able to interact physically with a model of the brain or skull. For example, when planning intracranial electrode implants for patients with epilepsy, interdisciplinary conferences often do not address the actual intended locations of the electrodes. Implantable electrode grids are often proprietary, deformable mesh devices, proving hard to model in 3D visualizations. A physical 3D model would allow clinicians to take devices and lay them on the brain and/or skull to plan surgical coverage, complementing information obtained by 2D or virtual 3D imaging. Additionally, physical 3D models may be more advantageous for clinical education than computer-based 3D models [1].

3D printing of physical objects has rapidly become both a source of novel biomedical and clinical applications as well as a cost effective means of producing 3D objects [2,3]. For example, 3D printing can be used to print implantable hard [4–6] and soft [7] tissue prostheses, to print tissue biomimetically [8,9], or to develop molds for tissue engineering [10–12]. Underlying many of these applications is the goal of patient-specific interventions. Patient-specific interventions may include nonsurgical purposes such as radiation planning [13], pre-operative planning [14,15], patient counseling [16], or the opportunity to practice complex procedures in specific clinical cases [17]. Some of the more complex applications use high-end industrial printers that can cost tens to hundreds of thousands of dollars, with material costs for printed objects of several thousand dollars [17]. While the expense may be necessary for some purposes, this cost can be prohibitive for different patient-specific applications, which may be amenable to less expensive 3D printing technology.

The progression of open source platforms has increased the capabilities and popularity of desktop 3D printers. These 3D printers are less expensive than industrial 3D printers with the most affordable and common type currently utilizing fused deposition modeling (FDM) technology, also known as fused filament fabrication (FFF). This additive process prints the object one layer at a time by extruding heated plastic through a nozzle, while moving around a build plate. Studies have already reported that models of some human organs can be printed relatively accurately from 3D printers [18–20], however, most studies lack detailed and generalizable methods for 3D printing that can be applied to patient-specific routine clinical imaging data. Here we describe our step-by-step, end-to-end process for generating and printing 3D models of patient specific human brains and skulls in a detailed, easily replicated methodology. Our process is simple, inexpensive, and mostly unsupervised. In addition to describing our experiences printing our 3D brain and skull models on open-source desktop 3D printers we present data on how to adapt this process to other desktop 3D printers. We additionally contextualize the possible utility of such a process through surveys with healthcare providers and patients in an epilepsy clinic.

## Materials and Methods

This study was approved by the Partners Human Research Committee Institutional Review Board (IRB), protocol 2007P000165. Written informed consent was obtained from the participants and the IRB approved the consent procedure, including review of patient imaging. The full workflow for our process is summarized in Table 1.

**Table 1:** Overall modular workflow described in the text for *A*. *Transforming 2D images to 3D models* and *B*. *Desktop 3D Printing* brains and skulls. We highlight, in each table cell, software or hardware that we have found produces reliable prints, noting the slight variations in our workflow process for MRI based brain models compared to CT based skull models. The modular nature of the workflow process allows for alternative methods to be used at each step. Each step and potential alternatives are described more thoroughly in the text. Step *A4*. *Crop 3D model* is italicized because this step is optional, primarily for use when one only needs to print a subset of the organ of interest.

A. Transforming 2D images to 3D models				B. Desktop 3D Printing		
1. Obtain radiology images	2. Create anatomical surface	3. Convert surface to 3D STL file	*4*. *Crop 3D model (optional)*	1. Generate gcode for 3D printing	2. 3D Print	3. Clean print
Brain MRI: KPACS	FreeSurfer: pial surfaces (3–5 hrs)	FreeSurfer or Matlab (<5 min)	*Blender (<15 min)*	ReplicatorG (40–60 min)	Flashforge CreatorPro (9–11 hr)	File & pliers (<30 min)
Skull CT: KPACS	InVesalius: selected bone (<30 min, outputs STL file)		*Blender (<15 min)*	ReplicatorG (20–40 min)	Flashforge CreatorPro (4–13 hr)	Dremel & pliers (<30 min)

### Creating Brain Surface

**Fig 1:**
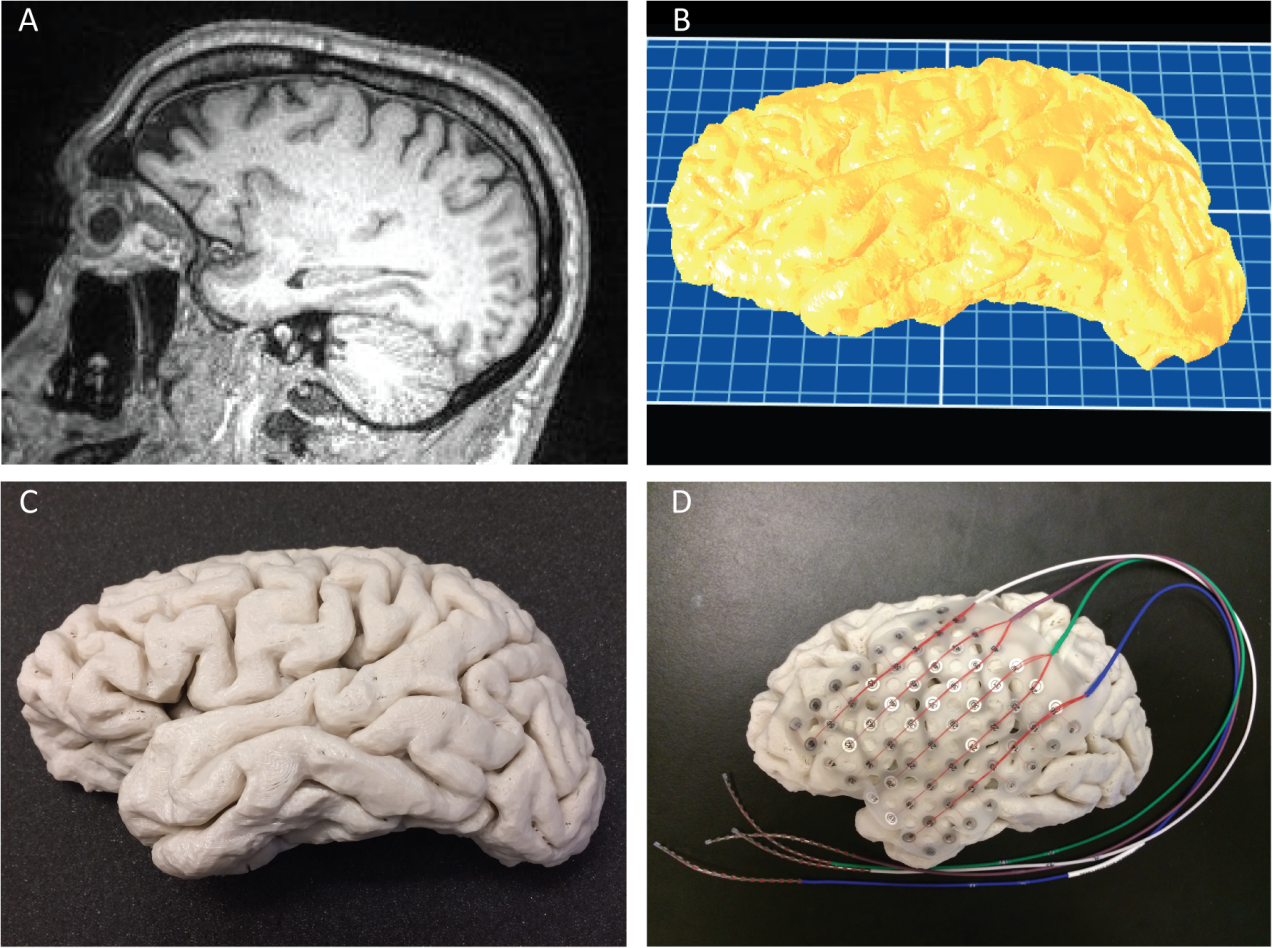
Brain Print Process. A) Sagital view of patient’s MRI (step A1). B) Brain surface rendered in ReplicatorG at the time of gcode generation (step B1). C) 3D printed brain after manually removing the support material (step B3). D) 3D printed brain overlaid with a 64-contact electrode grid to highlight possible electrode coverage during neurosurgical planning.

We use a patient’s brain MRI to construct the corresponding brain surface. First, we obtain clinical neuroimaging Digital Imaging and Communications in Medicine (DICOM) images using K-Pacs (http://www.k-pacs.net/), a DICOM visualization and downloading software (Table 1: step A1). Often, DICOMs can be obtained directly from the radiology department. We preview the images to ensure quality and resolution of the necessary sequences (T1-weighted 1 mm isotropic MPRAGE images) (Fig 1A). We convert the DICOM images into a model of the brain's pial surface using the recon-all function in FreeSurfer (http://surfer.nmr.mgh.harvard.edu/, free and open-source) (step A2). This function generates a pial surface for each hemisphere automatically and takes 3–5 hours (mean 3.57 hr, standard deviation 1.24 hr, n = 32 subjects) on a desktop computer (Dell XPS 8700, CPU Core i7-4770 @ 3.4 GHz, 12 GB RAM, GeForce GTX 645, running Debian Jessie [21]) using the GPU assisted version of recon-all [22].

We then convert the brain surface model into a standard 3D file format (STereoLithography file,.STL) (step A3). STL files can be edited in several open source programs (e.g. Blender, described below in optional step A4), transmitted to commercial 3D printing services, or translated into gcode for printing on a desktop 3D printer (step B1 described below). For the desired brain hemisphere, we load the pial surface into Matlab using freesurfer_read_surf (http://eeg.sourceforge.net/doc_m2html/bioelectromagnetism/freesurfer_read_surf.html) and export the STL file using the stlwrite function (http://www.mathworks.com/matlabcentral/fileexchange/20922-stlwrite-filename—varargin-). While we typically use Matlab to convert the FreeSurfer output to STL format, FreeSurfer output can be converted to STL files directly using the mris_convert function in FreeSurfer (http://freesurfer.net/fswiki/BlenderModel). For testing purposes, sample brain and skull STL models are also available for download from the NIH 3D Print Exchange (http://3dprint.nih.gov/).

### Creating Skull Surface

**Fig 2:**
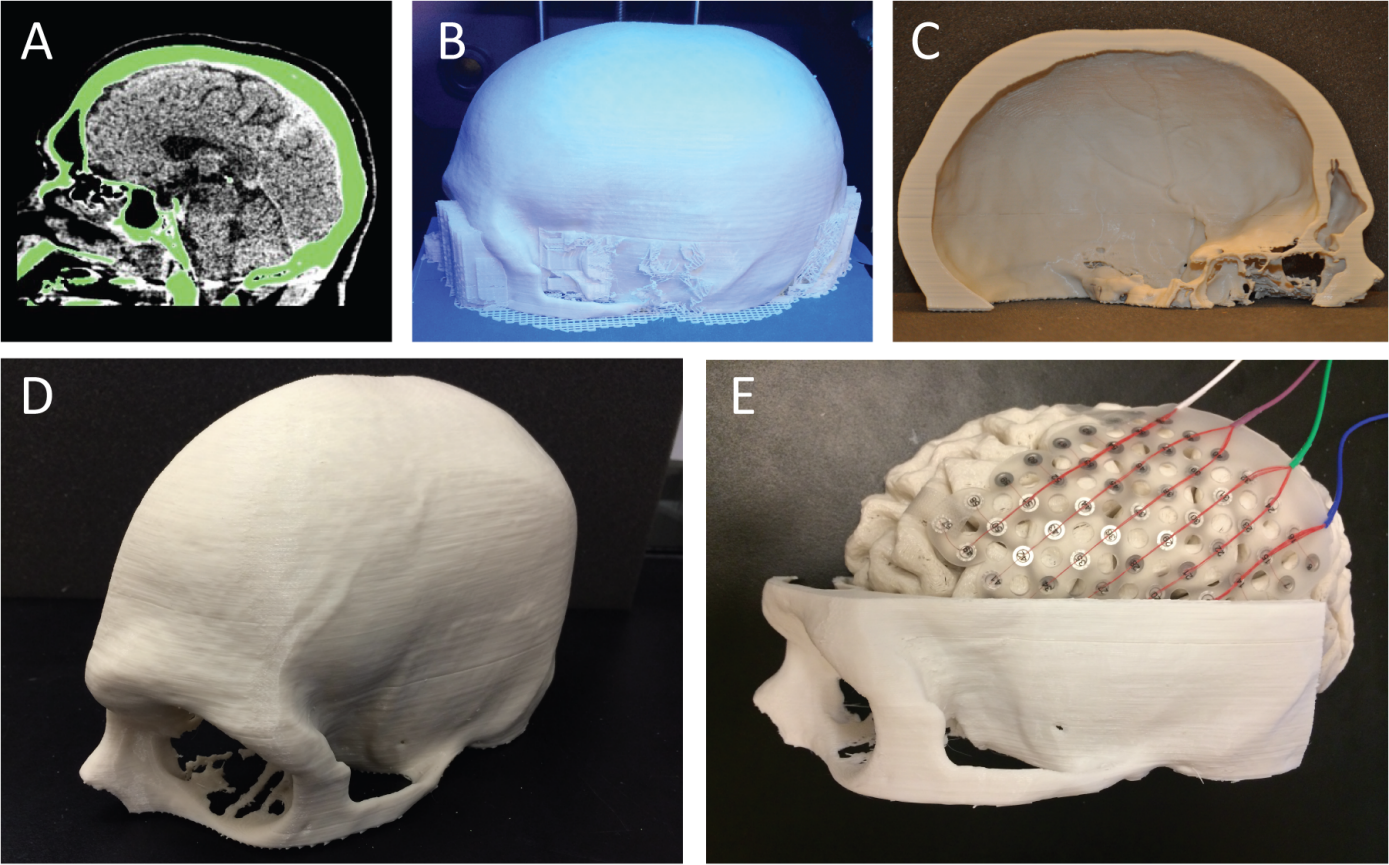
Skull Print Process. A) CT scan visualized using InVesalius. The green highlights the skull to be rendered, selected using image intensity (step A2). B) 3D printed skull with structural support from lateral viewpoint (step B2). C) 3D printed skull from medial viewpoint after removing the internal support material (step B3). D) 3D printed skull from anterolateral viewpoint after removing the support material. E) Anterior and inferior portion of skull and brain interlocking with a 64-contact electrode grid oriented for surgical planning.

We use the patient’s head CT to construct the skull rendering. Again, we use K-Pacs to obtain the DICOMs of the scan (step A1). Then we load the data into InVesalius (http://svn.softwarepublico.gov.br/trac/invesalius, free and open-source) and use image intensity to separate bone from non-bone (Fig 2A) and export an STL file (step A2-3).

1. Select the corresponding DICOMs.
2. Create a new mask using an intensity range that best isolates the skull from the rest of the scan (example range: 177–3071).
3. Configure the 3D surface by creating a new 3D surface.
4. In the advanced settings, select the largest surface. This will eliminate any stray artifacts from the scan and clean up the image.
5. Export the 3D surface as an STL file, for possible editing in Blender (step A4) or to advance to printing (steps B1-3).

Preprocessing CTs usually takes less than 1 hr total, including the optional Blender editing described next (step A4). Other possible open-source pre-processing software include ITK-SNAP (http://www.itksnap.org) [16].

### Editing STL files (Optional)

The STL outputs from step A3 can be used for 3D printing; however, if only a subset of an object is needed, then the STL file can be cropped in a 3D modeling program (step A4). We import the desired STL files into Blender v2.73a (http://www.blender.org/, free and open-source), a 3D modeling and rendering program. We ensure that the Editor Type: Properties: Units are set to *None* and *Degrees*. Units of *None* defaults to millimeters, which are the same units as the STL files used for 3D printing. A Boolean add-on (https://github.com/vitorbalbio/code/tree/master/BoolTool) simplifies the keystrokes involved in object editing. At this point, if we wish to crop an object, for example to highlight certain regions and/or ensure the print fits on a desktop build plate, we take the following steps (described for a *Skull*):

1. Add a *Plane* mesh into the scene
2. Orient the *Plane* so as it intersects with the *Skull* mesh where we choose to crop.
3. Select both the *Plane* and the *Skull* meshes in object mode and use the Boolean modifiers to crop. Note that the order in which you select the two images is important. We select the *Plane* first, then the *Skull*, and then use the Difference Boolean modifier. Sometimes this modifier selects the other side of the object than is desired. In this case, we rotate the *Plane* 180 degrees so that the normal vector is flipped. To do this,
  - Orient the screen so that the *Plane* is seen as a vertical line
  - Press **R, 180** to rotate 180 degrees perpendicular to the screen. We usually only crop skulls directly along the X, Y, or Z axis, making the process of flipping the normal vector of the *Plane* simpler.

For all objects, brain and skulls, we orient the mesh to optimize the location on the build plate of the printer. Use **NumPad 1**, **NumPad 3**, and **NumPad 7** to rotate the view and align the object along the axes. Also use **NumPad 5** to go to Orthographic view (in contrast to Perspective View), which creates gridlines at preset measurements. Using the grid as a reference, we move the object by pressing **G** so that it straddles the X an Y axes evenly. We try to place the object with the most inferior surface at Z = 0, but this can be automatically adjusted when creating machine instruction gcode (step B1 below). Many of these final positional steps can also be performed in ReplicatorG or other slicing programs (step B1). For the specified brain hemisphere, we usually print the medial surface facing down on the build platform because it is of least clinical interest for our surgical planning purposes. In general, the facedown surface will require the most post-processing, allowing users to select which face is least important for a given needs. For the skull, we usually print the inferior surface face down (similar to anatomical orientation). The total process of editing an STL file usually takes less than 20 minutes.

### Generating gcode

The STL files resulting from steps A1-4 (Transforming 2D images to 3D models) can be transmitted digitally to a commercial 3D printing service. However, we also print models on our own desktop 3D printer (steps B1-3). To do so, we convert the STL files into gcode, a programming language used for controlling automated machines such as desktop FDM/FFF 3D printers (step B1). We use ReplicatorG (http://replicat.org/, version 0040, free and open-source) to translate our STL files into gcode files. ReplicatorG can use several engines to generate gcode and we use the included Skeinforge engine (version 50). Skeinforge requires Python (http://www.python.org/), as described in the installation instructions for ReplicatorG (http://replicat.org/installation). We have successfully used the 32 bit versions of both Python 2.6 and 2.7.9 on a 64 bit Windows 7 installation. Other open-source alternatives for generating gcode for desktop FDM/FFF 3D printers include Slic3r and Cura. Using ReplicatorG, we import the STL and move the object to the build platform. This is a good time to adjust the final object orientation if desired. One can use the move tab to automatically "Center" the object or "Put on platform". When the object is aligned, select "Generate GCode".

When generating gcode, one can set parameters to determine how the model will be printed, such as layer thickness or feedrate. Specific parameter types depend on the type of 3D printing technology used. Most desktop 3D printers, especially those large enough to print brains and skulls, use fused deposition modeling (FDM)/ fused filament fabrication (FFF) as their primary printing process, melting and extruding plastic filament through a small nozzle. All the 3D printers we tested (described next in step B2) used this printing process. We performed a systematic exploration of all parameters available in ReplicatorG, which are similar to parameters in other slicing programs for desktop 3D printers. We printed brain regions using at least two different settings for more than ten different parameters, with at least two repetitions of the most reliable setting, yielding over 50 prints. In Table 2, we identify the parameters and values that yielded the most pragmatic trade-off between speed and reliability for our 3D printer in our environment. Given that different printers and different environments can require slight adjustments of these parameters, particularly depending on the purpose of the print, we also provide information on how to adjust parameters from these suggested starting points empirically in Table 2. In general, slower prints are likelier to be more successful; however, most failures will be evident within the first few layers, allowing for early empiric parameter adjustment even when printing rapidly.

**Table 2:** Suggestions on how to choose printing parameters for fused filament fabrication (FFF)/fused deposition modeling (FDM) 3D desktop printers. Different 3D printers and print environments may have subtle effects on the values of parameters needed in the workflow (step *B1*. *Generate gcode for 3D printing*). We therefore present the results of systematic parameter exploration (over 50 prints), to assist in empirically choosing and adjusting 3D printing parameters. Where noted, parameters may involve a tradeoff between speed and smoothness of the print, the choice of which may depend on the use of printed object. In parentheses we include the parameters that we have found to produce usable objects in the time needed (<1 day) reliably.

Parameter	Characteristics for reliable prints	If parameter is too low	If parameter is too high
**Build Plate Leveling**	Extruded filament is flattened, consistently adhering with an even height over the whole build plate (Gentle grip on piece of paper)	Filament will be overly round and adhere poorly to build plate. Print may curl or warp upwards.	Filament may extrude minimally or inconsistently, building up in the nozzle. The motor may click if there is too much resistance to flow.
**Filament selection/quality**	Filament width and adherence to plate and other layers is consistent, especially on support layers (StellarLabs PLA filament)	Filament width and adherence to build plate and other layers may be too variable, causing the filament to curl up and the print to fail.	n/a
**Extruder Temperature**	Smooth filament extrusion with flow primarily during printing (210°C)	Filament will not melt and extrude, and motor may click if this causes too much resistance to flow.	Filament will extrude during the preparatory printer warm-up, before the actual printing begins. Higher temperatures also presumably increase the risk of a fire hazard.
**Build Plate Temperature**	Good adherence of the print to build plate, but with relatively easy removal of the object from build plate at the end of printing (50°C)	First layers will not adhere well to the build plate, curling up and possibly causing the print to fail.	Object will adhere too strongly to the build plate, making it hard to remove at the end. Additionally, the first layers may discolor and widen out slightly on the build plate.
**Shells**	Appropriate balance between overhang support and print time, reflecting the purpose of object (1 shell)	Overhanging elements may be likelier to droop, but fewer shells yield a faster print time with less filament use.	More shells decrease drooping of overhanging elements and make the object more resilient to aggressive post-processing, but at the expense of increased print time and filament use.
**Infill**	Appropriate balance between resolution and print time, reflecting purpose of object (2% brains, 5% skulls)	Less infill yields a faster print time with less filament use, but the final object is likelier to be more fragile. Less internal support can cause also some holes on flatter, internally overhanging higher layers.	More infill increases print time and filament use when there is significant internal volume to the object. These costs affect brains more than skulls, due to the thinner nature of the skull volume. Final objects with greater infill are likelier to be sturdier, with more support for flatter higher layers that have an internal overhang.
**Feedrate/Travel speed**	Appropriate balance between resolution and print time, reflecting purpose of object (70 mm/s, 100 mm/s)	Higher print precision, slower print	Faster print, but decreased print precision. If too fast, filament extrusion may become variable and not adhere to prior layers. Fast speeds may also cause misalignment between layers and may cause the printer to vibrate excessively.
**Raft & Support**	All elements supported with easily removed external supports (Raft + External Support)	Printing without supports may cause overhanging elements to print in the air and/or adhere to nozzle, possibly causing the print to fail.	Full supports increase printing time, filament use, and post-processing time to clean and remove the supports.
**Layer Height**	Appropriate balance between resolution and print time, reflecting the purpose of object (0.3mm)	Smaller layer heights yield a higher print resolution, but slower print. The lower limit is limited by printer specifications.	Larger layer heights lower the print resolution, but yield a faster print. The maximal value is limited by printer specifications

We use the following settings when generating gcode for the FlashForge Creator Pro open-source printer described next in step B2:

Slicing Profile: Replicator slicing defaults
We use either Left or Right extruder, depending on where the filament has been loaded
Use support material: Raft & Exterior support
Use default start/end gcode
Use Print-O-Matic (stepper extruders only)
Object Infill (%): 2% brain, 5% skull
Layer height (mm): 0.3
Number of shells: 1
Feedrate (mm/s): 70
Travel Feedrate: 100
Print temperature (°C): 210
We do not modify the following printer-specific default settings on subsequent tabs:
Plastic: Filament Diameter (mm): 1.82
Extruder: Nozzle Diameter (mm): 0.4

Following the creation of the gcode file when printing with PLA plastic, we also change the heated build plate temperature setting to 50°C, by changing the following line in the text file under the gcode tab:

M109 S**110** T1 (set HBP temperature)-> M109 S**50** T1 (set HBP temperature)

For brain hemispheres, generating gcode takes approximately 50 minutes (mean 52.41 min, standard deviation 8.29 min, n = 8 subjects) on a desktop computer (same hardware specifications as above but with an integrated rather than dedicated GPU, running Windows 7, 64-bit) (example in Fig 1). For skulls, in which we often crop the region of interest depending on the intended use, gcode generation can be more variable depending on the size of the printed portion, but takes approximately 30–40 minutes for skull "hemispheres" (example in Fig 2B–2D).

### 3D Printer and Filament

We have successfully printed our designs on both a FlashForge Creator Pro (released in 2014, currently $1,199) and a Makerbot Replicator (original model released in 2012 with dual extruders, $1,999). These printers are based on the open-source Rep-Rap design (http://reprap.org). Given that the original Makerbot Replicator is no longer supported by Makerbot, and newer versions of the Makerbot Replicator are no longer open-source, we focus primarily on our printing experiences with the FlashForge Creator Pro.

We have printed our models using both polylactic acid (PLA) and acrylonitrile butadiene styrene (ABS) plastics with similar results overall. In general, PLA consistently warps less than ABS and contains fewer split layers when printing more rapidly; therefore we print primarily in PLA. PLA also tends to be more widely used as it does not necessarily require a heated-build plate. There are many suppliers of PLA filament and we have tested multiple colors of filament from 3 different manufacturers (Stellar Labs, FlashForge, and Makerbot Industries). We have had success using PLA filament manufactured by Stellar Labs and distributed by MCM Electronics. We have tested 4 different colors of this filament: Arctic White, Battleship Gray, Sonic Silver, and Midnight Black. Although different colors of plastic may in principle require slightly different temperatures for optimal use, we have been able to print with each of these colors using the settings above and have observed that the variability between manufacturers is usually more significant than variability between different filaments from the same manufacturer for our prints. Both printers above use 1.75mm diameter filament. At the time of writing, a 1 kg spool of 1.75mm Stellar Labs PLA filament costs $19.99 (via MCM Electronics)

### Pre-print printer inspection

#### Build-plate surface

For PLA printing, we cover our build plate with blue painter's tape that came with our FlashForge Creator Pro. This is standard blue painter's tape available from most hardware suppliers (e.g. 3M #2090, Blue Long-Life Masking Tape, available from McMaster). The FlashForge Creator Pro also ships with a layer of polyimide tape (also known as Kapton) on the build plate. We have printed with and without this layer of polyimide tape, placing the blue painter's on top of it or directly on the build plate, with similar results. Polyimide tape is more commonly used for ABS plastic prints, and we have found it necessary in order to adhere ABS prints to a hotter build plate (110°C). Not all desktop 3D printers have heated build plates; therefore PLA filament is likelier to be a more widely usable filament for this reason as well.

#### Modified filament tube holder

We have modified the FlashForge Creator Pro filament tube holder using a user contributed substitute (http://www.thingiverse.com/thing:409297). This design holds the filament tubes slightly further away from the back of the printer than the standard tube holders. While we did not have any initial problems with the standard configuration of the FlashForge Creator Pro, we did have problems with the Makerbot Replicator tube holders being too close to the printer and straining the plastic filament. We therefore preemptively incorporated this tube holder modification for the FlashForge Creator Pro.

#### Leveling the Build-plate

After several prints we re-level the build plate by checking the distance between the extruder nozzle and the build plate using a piece of paper, making sure there is a gentle grip at all portions of the plate (Table 2). We additionally check leveling by observing the consistency and thickness of the extruded plastic as the first layer prints, to ensure that plastic extrudes in a smooth, flat layer. Interrupted extrusion suggests that the extruder nozzle and build plate are too close together; round extruded filament that does not stick to the build plate or successive layers suggests that the extruder nozzle and build plate are too far apart (Table 2). There are numerous online tutorials and examples of how to level a build plate (e.g. http://makerbot.wikia.com/wiki/Build_Platform_Levelling).

### Post-print cleaning

The printed objects are described below in the Results. However, following printing, most of our 3D models contain support plastic that must be removed, a process that takes about 20 minutes to do so manually using needle-nose pliers and safety goggles. The plastic supports on the bottom of the print, i.e. surface of the print in contact with the build plate, tend to be more difficult to remove. We have tried to clean this area using sand paper, files, heat guns, and dremel grinding bits (Grinding Bit 1/8" Shank, 1/4" Head Diameter, 5/16" Head Length, B43, McMaster 4522A169), while wearing a respirator face mask. In general, cleaning this surface to a pristine condition is not always time efficient, and we therefore print our objects with the least important surface for our purposes face down. We usually post-process the smaller, tighter curves of the brain prints using a combination of files and needle-nose pliers, whereas we post-process the larger, smoother curves of the skull prints using a dremel grinding bit and needle-nose pliers. The dremel works best when using it at a low speed, so as to avoid melting the plastic. Edges of the print and supports may be sharp and care should be exercised. We have found that ABS prints may be slightly easier to clean, and one can consider using an acetone vapor bath with ABS prints to smooth the surface; however we tend to print in PLA for the reasons described previously. Post-processing can also be simplified by using dissolvable plastic filament, however, the reliability of dissolvable filaments on inexpensive desktop 3D printers remains unclear.

### Measuring Print Accuracy and Quality

Following cleaning, we measure the object's maximum dimensions in three defined axes using electronic calipers (e.g. McMaster, 4996A18) to compare the print size with the patient's original imaging. We focus here primarily on our measurements of brain prints, given that skull print accuracies have been reported previously [23,24]. Because of the possible ways in which the brain can be rotated at the time of imaging, one can not assume that the clinical images are in the same plane as the brain print, particularly for coronal and axial images. Therefore, we measure maximal distances between identified brain points, which remain consistent with rotation. For an anterior-to-posterior measurement, we measure the maximal distance between the most anterior portion of the frontal pole and the most posterior portion of the occipital pole, parallel to the medial surface of the brain hemisphere. For a medial-to-lateral measurement, we measure the maximal distance perpendicular from the midline to the most lateral portion of the temporal lobe. For a superior to inferior (S-I) measurement, we measure the maximal distance between the most superior portion of the motor strip and the most inferior portion of the temporal tip. Measurements were obtained jointly by two evaluators.

The primary purpose of determining the quality of the print for our needs depends upon their functional context [20], i.e. whether one could lay an implantable electrode grid on the surface without significant deviation. In order to grade the quality of the prints, we use a structured rubric (S1 Table). The rubric divides prints into *Good*, *Fair*, and *Poor* prints. *Good* represents aesthetically and functionally satisfactory prints. *Fair* represents prints with minor aesthetic imperfections that do not significantly impact the function of the print. *Poor* represents prints that contain deviations that are likely to be unsatisfactory for use. An overall print grade is determined by the lowest partial grade, i.e. a print must attain a grade of *Good* in all three qualities to be considered *Good* overall. The details of this rubric are presented in S1 Table. All prints were graded by two separate reviewers.

### Surveys

We surveyed healthcare providers and patients associated with our epilepsy service to determine whether personalized, patient-specific 3D printed models might improve interdisciplinary care and patient education. Healthcare providers participating in our institutional interdisciplinary epilepsy surgical conference were shown a 3D printed model of a brain hemisphere from a patient being considered for surgery and asked "Do you think that personalized, 3D printed models like this one may be helpful in the epilepsy surgical planning conference?". Responses were collected anonymously and choices were *Extremely helpful*, *Very helpful*, *Somewhat helpful*, *Not at all helpful*, *Unhelpful*. Patients who attended a session of our epilepsy clinic were approached to see if they would participate in a brief survey. Participants were shown a 3D printed model of a brain hemisphere from an anonymous clinic patient, and asked "Do you feel that personalized, 3D printed models like this one could help you understand the care we provide?". Response choices were *Extremely helpful*, *Helpful*, *Somewhat helpful*, *Not at all helpful*, *Unhelpful*.

## Results

We have printed 19 brain hemispheres (example in Fig 1), over 30 brain regions, and 7 skulls (example in Fig 2). Details of three different brain hemispheres are given in Table 3. Despite the varying sizes of the patients' hemispheres, print dimensions were within 1–2 mm of MRI measurements (mean difference 0.5 mm, standard deviation 0.3 mm, maximum difference 1.2 mm). This represents overall size discrepancies of <1% (mean 0.54%, standard deviation 0.43%), similar to prior reports for other structures [23]. The regions most likely to be affected by poor resolution were the smallest structures, such as thin portions of the skull, also consistent with prior reports [25]. To determine the consistency of prints, we printed the same patient-specific hemisphere five different times. Across repeated prints of the same hemisphere (Subject C), print dimensions were within 1 mm of each other (standard deviation of 0.36–0.55 mm). Prints could be used empirically to help determine appropriate electrode coverage with implantable electrode grid meshes (Figs 1D and 2E).

**Table 3:** Measurements of brain hemispheres from three different patients (in mm). The size of the final cleaned print is given along three axes: anterior to posterior (A-P), medial to lateral (M-L), and superior-to-inferior (S-I), measured as defined in the Methods. Across all prints, the difference between the print and MRI measurements are typically less than 1mm (mean difference 0.5mm, standard deviation 0.3mm, maximum difference 1.2mm). For subject C, we printed the same hemisphere five different times in order to determine the consistency of prints. Across repeated prints of the same hemisphere (Subject C), prints were within 1mm of each other (standard deviation of 0.36–0.55mm).

**Subject A**	**A-P (mm)**	**M-L (mm)**	**S-I (mm)**
**Print**	163.50	62.98	114.28
**MRI**	164	62	114
**Subject B**	**A-P (mm)**	**M-L (mm)**	**S-I (mm)**
**Print**	189.20	67.80	121.76
**MRI**	189	67	121
**Subject C**	**A-P (mm)**	**M-L (mm)**	**S-I (mm)**
Print 1	173.47	61.25	117.20
Print 2	173.25	62.38	116.30
Print 3	172.58	62.52	116.24
Print 4	173.43	61.83	116.25
Print 5	173.13	62.48	116.90
**Prints Mean**	173.17	62.09	116.58
Standard Deviation	0.36	0.55	0.44
**MRI**	173	62	116

The time and amount of plastic used with each print depends primarily on the volume of the object, the layer thickness, the number of shells, and the amount of infill (Table 2). Using the parameters identified above on the specified printer, a single brain hemisphere print takes 9–11 hours (Fig 1B–1D). The weights of brain hemispheres printed at the recommended settings, including supports prior to removal, are approximately 150–190 g, yielding filament costs of $3-4.The print times and weights for the skull depends on the overall object size determined by any relevant cropping. For example, printing a left half of the skull, with the inferior boundary determined by the external auditory meatus and the inferior orbital ridge, took 11 hr 53 min (Fig 2B–2D), yielding a weight of 223g and cost of $4.46. Printing only an inferior portion of this part of the skull with the superior margin determined by the superior orbital ridge and a posterior margin 3.5 cm from the external acoustic meatus took 4 hr 22 min (Fig 2E), yielding a weight of 60 g and a cost of $1.20. Reprinting the skull with a different orientation or portion requires significantly less preprocessing time because the original STL generated from the DICOMs can be reused (steps A1-3 do not need to be repeated), however one must regenerate the gcode (steps B1-3). All displayed prints are graded as *Good* according to our rubric for grading print quality by two different raters (S1 Table). In general, smaller prints at slower speeds have a higher likelihood of success. The stated parameters (Table 2) minimize the amount of printing time while maintaining *Good* quality prints (S1 Table).

At the time of this writing, we have most recently been using the FlashForge Creator Pro 3D desktop printer for several months. The process described above represents improved success rates over our previous use on a Makerbot Replicator desktop printer (~30–40% success for brain hemispheres, no attempted skull prints). The primary reason for previously failed prints were object warping using ABS filament (diminished by using PLA plastic) and “air prints”, when filament stops extruding for unclear reasons and is often difficult to resolve. We have not had these difficulties with the newer printer, except for when we purposely adjusted the printing temperature to be too extreme (Table 2), suggesting a significant improvement in printing technology in the intervening years. In addition to these printers, we have successfully printed a brain hemisphere with a commercial 3D printer distributor using a uPrint SE printer with an ABS plastic (manufactured by Stratasys, distributed by R&D Technologies). In this case, steps A1-3 to generate the model were the same, yielding a file that was sent to the distributor instead of desktop printing in house (deferring steps B1-3). The print was similarly successful with a several day turnaround and very good quality (material cost $80).

**Fig 3:**
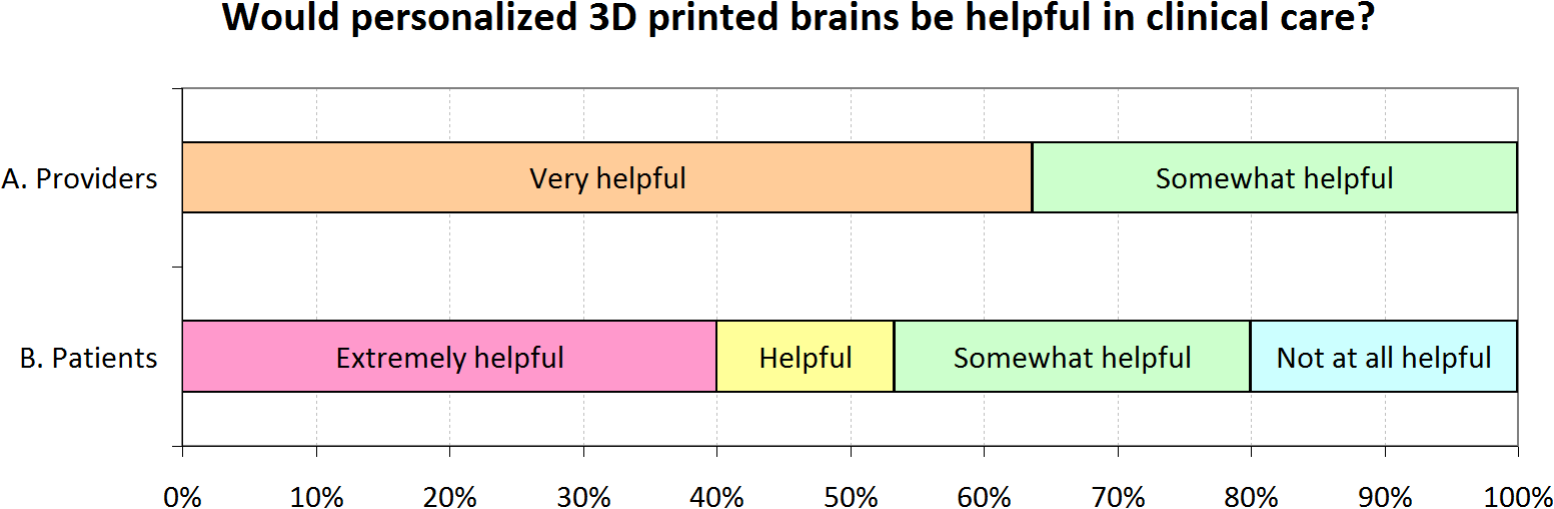
Survey Results. A) Healthcare Providers were surveyed as to whether personalized 3D printed brains would be helpful for interdisciplinary epilepsy surgical conferences. Of 11 respondents, 64% said that a personalized brain would be very helpful. B) Patients were surveyed as to whether a personalized 3D printed brain could help them understand their care. Of 15 respondents, 53% said a 3D printed brain would be helpful or extremely helpful.

We surveyed healthcare providers and patients associated with our epilepsy service to determine whether personalized 3D printed models might improve patient care and education (Fig 3). Of 13 providers invited to participate, 11 returned responses: 7 providers thought personalized 3D printed models would be a very helpful component of the epilepsy interdisciplinary conference and 4 providers thought they would be somewhat helpful. Of 17 patients invited to participate, 15 participated in the survey: 6 thought personalized 3D printed models would be extremely helpful in understanding their care, 2 thought they would be helpful, 4 thought they would be somewhat helpful, and 3 thought they would not be helpful. In summary, a majority of both healthcare provider and patient respondents thought that personalized 3D printed brains would at least be helpful, and no respondent in either group thought that the models would be unhelpful.

## Discussion

We have described here our streamlined and inexpensive method for 3D printing person-specific brains and skulls. Whereas several groups have presented the possible functions of 3D printed objects, we have presented more detail on a step-by-step process of how to go from clinical DICOM images to final 3D object, and provided data by which to select desktop 3D printing parameters as well as how to modify them for a given desktop 3D printer, environment, or need. Our method makes use of standard clinical or research neuroimaging data, which is then translated into models and machine instructions for 3D printing. This process is largely unsupervised and uses primarily free and open-source software whenever possible.

While much of our process is unsupervised, the majority of the time for printing a 3D brain model is taken up by two largely automatic steps: preprocessing of the high resolution imaging data in FreeSurfer and 3D printing. In our institution, because of multiple interdisciplinary studies, high resolution FreeSurfer outputs are available to us the day after a patient’s MRI, which is frequently well in advance of clinical and surgical discussions. Alternative software could also be used for preprocessing. For example, InVesalius can also be used for MRI segmentation, however, this requires increased user interaction in window selection and post-segmentation editing, therefore, decreasing overall processing time only through increased upfront user time. Some of our collaborators have reported using commercial software (BrainLab) that can convert DICOMs to STLs in less than 1 hour. The modular nature of the process we have described, however, allows for users to swap in alternative software or hardware for specific steps based on familiarity and availability.

The other major time cost is the duration of 3D printing (e.g. 9–11 hours for brain hemispheres). We have significantly decreased the time it takes to make prints from default settings (up to 24 hours) as a result of empiric parameter exploration (Table 2). Additionally, just as we have noticed a significant increase in printing success over just two years, we anticipate that upcoming desktop 3D printing technology will improve the speed of printing itself [26]. One does not necessarily need to purchase a 3D printer to print these objects. The models encapsulated in the STL files can be outsourced to commerical print services (sent after step A, deferring step B). However, by printing in-house (steps B1-3), we can complete our process for even complex individual models on the day after the neuroimaging scans without any delays due to shipping. This also obviates any potential HIPAA or privacy concerns with sending person-specific models outside of a clinical or academic institution. The cost of a 3D printer can also be amortized over the number of planned brains, as well as any other objects that will be 3D printed for other purposes.

3D printing is rapidly changing the landscape of research, education, and clinical practice. Producing an actual object that can be held in one's hands has significant functional advantages that complement standard 2D representation of imaging data and even 3D computer-based virtual models. In the context of surgical planning, one can lay implantable devices on the skull or brain to see precise ultimate spatial fits, as well as anticipate surgical approaches such as any bone windows. The 3D models can also be excellent educational tools that are more robust and less toxic than fixed tissue [27–29]. Other groups have developed more varied materials to help simulate and practice various basic and advanced surgical procedures, including third ventriculostomies [17] and spinal injections [30]. In addition to CT and MRI, other imaging modalities such as ultrasound can be used to create patient-specific 3D models [31]. We have also used printed models in discussions with patients, to help illustrate in a patient specific manner the various procedures that will be performed, and have received positive feedback and appreciation from this patient engagement. Given the individually focused nature of many complex medical and research activities, streamlined and inexpensive 3D printing allows us to hold the objects of our attention in both the hand and mind's eye simultaneously.

## Supporting Information

S1 Table Rubric for judging quality of 3D printed models. The primary purpose of determining the quality of the print is whether one could lay an implantable electrode grid on the surface without significant deviation. *Good* represents aesthetically and functionally satisfactory prints. *Fair* represents prints with minor aesthetic imperfections that do not significantly impact the function of the print. Poor represents prints that contain deviations that are unsatisfactory for use. An overall print grade is determined by the lowest partial grade, i.e. a print must attain a grade of *Good* in all three qualities to be considered *Good* overall. All prints displayed in Figs 1 and 2 were graded as *Good*.
(DOC)
