# Supplementary material for: Streamlined, Inexpensive 3D Printing of the Brain and Skull
Source: PLoS One. 2015 Aug 21;10(8):e0136198. doi: 10.1371/journal.pone.0136198 (PMC4546422; doi:10.1371/journal.pone.0136198)
Supplement: S1 Table — The primary purpose of determining the quality of the print is whether one could lay an implantable electrode grid on the surface without significant deviation. Good represents aesthetically and functionally satisfactory prints. Fair represents prints with minor aesthetic imperfections that do not significantly impact the function of the print. Poor represents prints that contain deviations that are unsatisfactory for use. An overall print grade is determined by the lowest partial grade, i.e. a print must attain a grade of Good in all three qualities to be considered Good overall. All prints displayed in Figs 1 and 2 were graded as Good. (DOC) [file pone.0136198.s001.doc]

**Streamlined, inexpensive 3D printing of the brain and skull**

**Supporting Information**

Jason S. Naftulin^1¶^, Eyal Y. Kimchi^1,2*¶^ and Sydney S. Cash^1, 2^

^1^Department of Neurology, Massachusetts General Hospital, Boston, Massachusetts, United States of America

^2^ Harvard Medical School, Boston, Massachusetts, United States of America

^*^ Corresponding author

^¶^ These authors contributed equally to this work.

| **Feature** | **Good** | **Fair** | **Poor** |
| --- | --- | --- | --- |
| Possible holes on print surface | <2mm maximal diameter of holes | 2-4mm diameter holes that do not disrupt overall shape for grid placement | >4mm diameter holes |
| Sagging of model overhangs | ≤1 area with visible overhang sagging that is ≤1cm in length | 2-4 areas of sagging, all ≤1cm in length | >4 areas of >1cm length of any 1 area of overhang sag |
| Splitting between layers | ≤1 split, ≤1mm deviation | 1-2 splits, each ≤2mm deviations | >2 splits or >2mm deviations between layers |

**Supplementary Table 1** Rubric for judging quality of 3D printed models. The primary purpose of determining the quality of the print is whether one could lay an implantable electrode grid on the surface without significant deviation. *Good* represents aesthetically and functionally satisfactory prints. *Fair* represents prints with minor aesthetic imperfections that do not significantly impact the function of the print. Poor represents prints that contain deviations that are unsatisfactory for use. An overall print grade is determined by the lowest partial grade, i.e. a print must attain a grade of *Good* in all three qualities to be considered *Good* overall. All prints displayed in Figures 1-2 were graded as *Good*.
